# Assessing the contribution of interferon antagonism to the virulence of West African Ebola viruses

**DOI:** 10.1038/ncomms9000

**Published:** 2015-08-05

**Authors:** Eric C. Dunham, Logan Banadyga, Allison Groseth, Abhilash I. Chiramel, Sonja M. Best, Hideki Ebihara, Heinz Feldmann, Thomas Hoenen

**Affiliations:** 1 Laboratory of Virology, Division of Intramural Research, National Institute of Allergy and Infectious Diseases, National Institutes of Health, 903 South 4th Street, Hamilton, Montana 59840, USA

## Abstract

The current Ebola virus (EBOV) outbreak in West Africa is unprecedented in terms of both its size and duration, and there has been speculation and concern regarding the potential for EBOV to increase in virulence as a result of its prolonged circulation in humans. Here we investigate the relative potency of the interferon (IFN) inhibitors encoded by EBOVs from West Africa, since an important EBOV virulence factor is inhibition of the antiviral IFN response. Based on this work we show that, in terms of IFN antagonism, the West African viruses display no discernible differences from the prototype Mayinga isolate, which corroborates epidemiological data suggesting these viruses show no increased virulence compared with those from previous outbreaks. This finding has important implications for public health decisions, since it does not provide experimental support for theoretical claims that EBOV might gain increased virulence due to the extensive human-to-human transmission in the on-going outbreak.

## Abstract (web summary)

Concerns have been raised regarding potential increase in the virulence of the Ebola virus during the current West African outbreak. Here the authors show that the ability to inhibit interferon response, one of the aspects of Ebola virus virulence, does not differ between the prototype Mayinga and isolates from the current outbreak.

## Graphical abstract

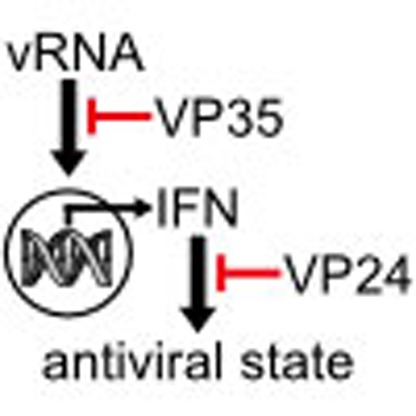

Ebola virus (EBOV) is a negative-sense, single-stranded RNA virus that causes a severe haemorrhagic fever in humans with high case fatality rates, ranging from 47 to 91% (ref. 1). In December 2013, an outbreak of EBOV Haemorrhagic Fever (EHF) began in Guinea^2^ and has since spread to become what is by far the largest and longest-lasting outbreak of the disease in history, having currently affected over 17,600 people (as of 12 June 2015; http://apps.who.int/ebola/en/current-situation/ebola-situation-report). Of particular concern is the unprecedented number of human hosts encountered by the virus, which has given rise to fears that it will exhibit changes in virulence or transmissibility through adaptation, although such changes have so far not been reported. Genetic variation is observed between the West African Makona variant viruses and those associated with previous outbreaks, although only a very limited number of mutations have been observed among sequenced samples taken from EHF patients during the current outbreak^2^,^3^,^4^. It is of great interest from both an academic and a clinical perspective to determine whether these mutations might have functional consequences for EHF pathogenesis, something that until now has not been addressed experimentally at the molecular level.

Although virulence in EBOV infection is certainly multifactorial in nature and has yet to be fully understood, in several instances, including animal models, it has been shown to be correlated with antagonism of the type I interferon (IFN) response^5^,^6^. We therefore investigated the IFN antagonism of the EBOV proteins VP35 and VP24 from Makona variant viruses. VP35 and VP24 have been shown to have activity in human cells as antagonists of IFN production and signalling, respectively^7^. VP35 strongly inhibits the production of both IFN-α and IFN-β^8^. Though the mechanisms for accomplishing this have yet to be fully described, it has been shown that interactions with both double-stranded RNA^8^,^9^ and the RIG-I signalling pathway, specifically by inhibiting the IRF3/7 kinases IKKε and TBK1 (refs 10, 11), contribute. Importantly, recombinant EBOVs with VP35 deficient in IFN antagonism are rendered non-lethal in guinea pigs^5^, supporting the idea that IFN suppression is critical to pathogenesis. In addition to its role in innate immune suppression, VP35 also serves as a polymerase cofactor, and is therefore essential for transcription and replication^12^.

VP24 likewise fills multiple roles during the viral life cycle. It regulates both transcription and replication^13^,^14^,^15^, most likely by contributing to condensation of nucleocapsids^16^,^17^,^18^. In addition to this, VP24 blocks IFN signal transduction by multiple means. It has been shown to interact with karyopherin-α^19^,^20^,^21^ and the heterogeneous nuclear ribonuclear protein complex C1/C2 (hnRNP C1/C2)^22^, while also blocking phosphorylation of p38-α^23^, all resulting in suppression of IFN signalling.

In this study, we examine several variants of VP35 and VP24 corresponding to the different genotypes of Makona variant EBOVs circulating in West Africa for their ability to inhibit the IFN response. Importantly, we find no apparent differences in the expression of these proteins in mammalian cells, their function in viral genome transcription and replication or their ability to inhibit the IFN response, which does not support a potential for increased virulence of EBOV Makona via this mechanism.

## Results

### Cloning and expression of VP35 and VP24 variants

**Figure 1:**
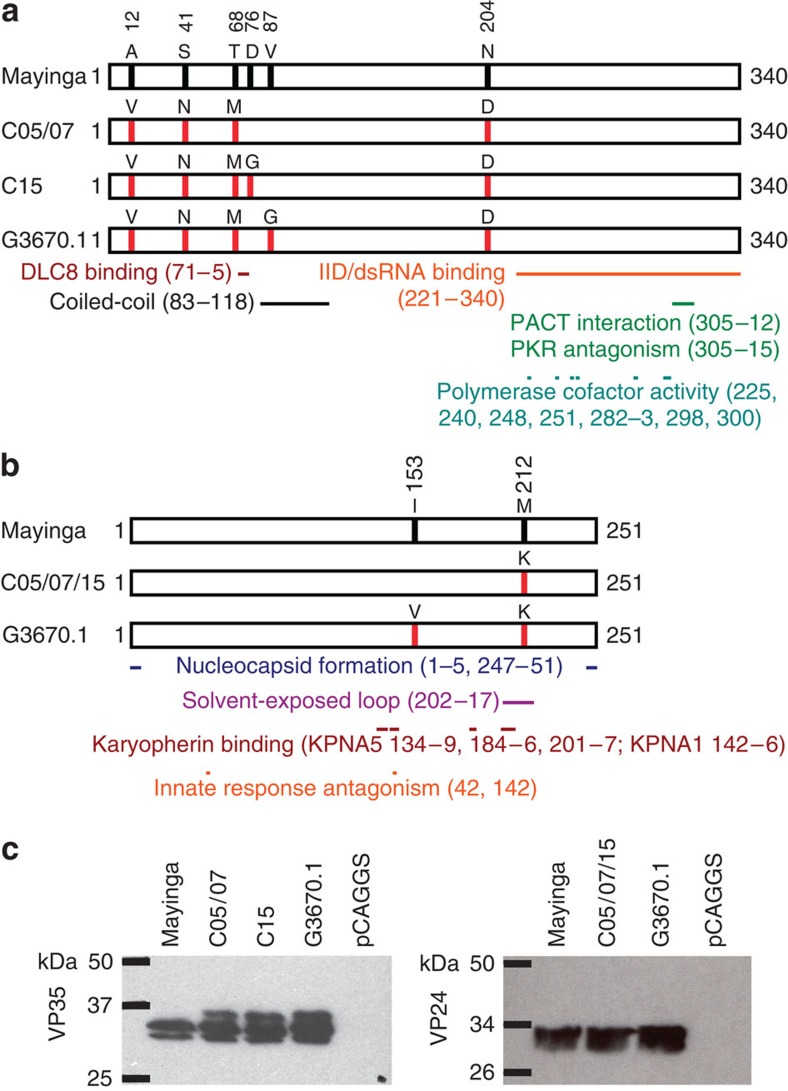
VP35 and VP24 variants used in this study. (**a**) Schematic diagram of VP35 variants used in this study. Labelled red vertical bars indicate residues where Makona protein sequences exhibit changes compared with Mayinga (May) reference sequence, where these positions are shown as black bars. Below the diagrams, coloured bars indicate known functional domains and regions of interaction with other proteins (DLC8 binding domain^28^, IFN inhibitory domain (IID)/double-stranded RNA (dsRNA)-binding domain^8^,^29^, coiled-coil domain^30^, PACT interaction domain^31^, PKR antagonism domain^32^ and polymerase cofactor activity^33^). Within the IID/dsRNA binding domain, amino acid residues R305, K309, R312, K319, R322 and K339 are involved in dsRNA binding^34^,^48^. (**b**) Schematic diagram of VP24 variants used in this study. Red and black bars within box diagrams represent mutations in Makona sequences compared with the Mayinga sequence, while coloured bars below diagrams represent functional domains (nucleocapsid formation^15^, solvent-exposed loop^35^ and karyopherin α1 (KPNA1) interaction domain^36^). Residues 42 and 142 are known to be important for innate immune response antagonist activity^19^,^34^. Residues that have been shown to be critical for KPNA5 binding are 134, 136–39, 184–86, 201, 203–05 and 207 (ref. 21). Amino acid sequences were aligned using the Clustal Omega software. (**c**) Relative expression levels of Makona variant VP35 and VP24 variants in human cells. 293 cells were transfected with expression plasmids encoding the four variants of VP35 or three variants of VP24 described above. Forty-eight hours later, cell lysates were subjected to SDS–PAGE and western blotting.

Based on multiple sequence alignments, we cloned all unique (on the amino acid level) VP35 and VP24 sequences found among the EBOV Makona variant genotypes published at the time of submission (that is, EBOV H.sapiens-tc/GIN/2014/Makona-WPG-C05, -C07 and -C15, hereafter referred to as Makona-C05, Makona-C07 and Makona-C15 (ref. 24), 99 genotypes from Sierra Leone^3^ and 4 genotypes from Mali^4^) (Fig. 1a,b). To compare the expression levels of these proteins, each construct was transfected individually into 293 cells and, following lysis and SDS–polyacrylamide gel electrophoresis (SDS–PAGE) protein levels were detected by western blot (Fig. 1c, Supplementary Fig. 1). We found that the new VP35 and VP24 constructs were expressed at levels approximately equal to VP35 and VP24 proteins from the prototype Mayinga isolate of EBOV (EBOV H.sapiens-tc/COD/1976/Yambuku-Mayinga). Interestingly, we observed multiple bands for VP35, with the highest molecular weight isoform not being present for Mayinga VP35. Whether this difference is due to alterations in post-translational modification is the subject of on-going studies.

### Analysis of VP35 and VP24 using a minigenome system

**Figure 2:**
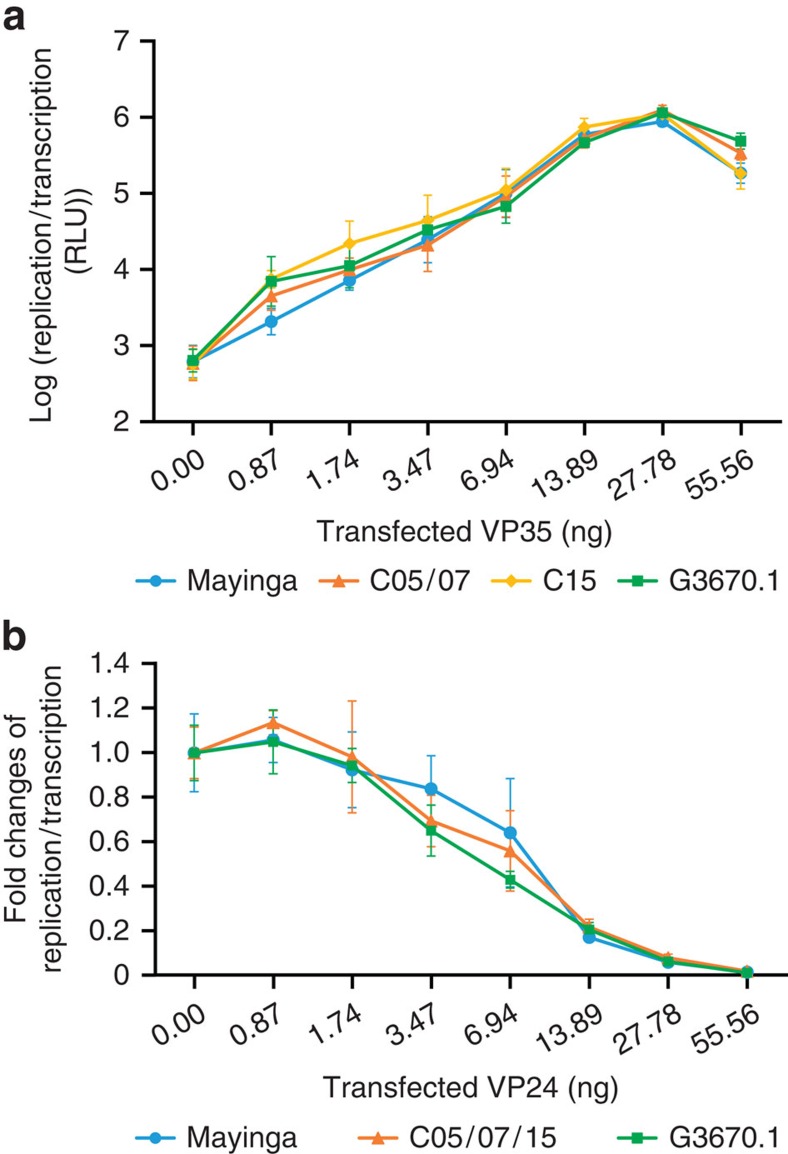
Function of VP35 and VP24 variants in replication and transcription. (**a**) Minigenome reporter activity in presence of VP35 variants. 293 cells were transfected with a monocistronic EBOV minigenome plasmid^12^ along with plasmids encoding the T7 polymerase and the EBOV proteins L, NP and VP30, as well as varying amounts of the different VP35 variants. After 48 h, cells were harvested and minigenome reporter activity reflecting viral genome replication and transcription was measured. The mean and s.d. shown represent six biological replicates from two independent experiments. Due to the large range of values, data are shown on a log scale. (**b**) Minigenome reporter activity with cotransfection of VP24 variants. 293 cells were transfected with an expression plasmid for EBOV Mayinga VP35 and the other plasmids as described for **a**. The different VP24 variants were coexpressed in increasing concentrations. After 48 h, cells were harvested and minigenome reporter activity was measured. The mean and s.d. shown represent six biological replicates from two independent experiments.

Our next goal was to determine whether the Makona variant VP35 and VP24 proteins had any altered effect on viral transcription/replication. To this end, we employed a monocistronic minigenome system, as described previously, to model viral genome replication and transcription^12^. In this assay the EBOV proteins NP, L, VP35 and VP30 are coexpressed in mammalian cells together with a miniature version of the viral genome, in which the viral genes have been replaced with a luciferase-reporter gene, but which still contains the genome termini^25^. Since these termini harbour the signals necessary for recognition as legitimate template by the viral polymerase, the minigenome is replicated and transcribed by the viral polymerase complex, resulting in production of reporter mRNAs, and ultimately reporter activity reflecting those processes. To test the ability of our different VP35 constructs to function as a cofactor for the viral polymerase, each construct was substituted into the minigenome assay in place of Mayinga VP35 at eight different concentrations. In these experiments, all of the VP35 variants showed effects on replication and transcription similar to the prototype Mayinga VP35 (Fig. 2a), and no significant differences could be detected.

To test the effect of VP24 as an inhibitor of minigenome transcription/replication, plasmids carrying each of the VP24 constructs were transfected at eight concentrations, in addition to the standard minigenome assay components as described above. Similar to the situation observed for VP35, these VP24 variants showed effects on transcription/replication similar to those for Mayinga VP24 over a range of protein expression levels (Fig. 2b), and no significant differences could be detected. Together with the VP35 data this suggests that the Makona variant-specific genetic differences have no impact on the functions of VP35 and VP24 in viral genome replication or transcription. This is despite the fact that one of the observed differences in the Makona variant VP35 (D76G in isolate Makona-C15) is adjacent to the domain recently shown to be responsible for binding to dynein light chain 8 (DLC8), an interaction involved in the regulation of genome replication and transcription^26^ (Fig. 1), although none of the other differences coincide with domains known to be involved with virus genome replication or transcription.

### Inhibition of the IFN-β response by VP35 and VP24

**Figure 3:**
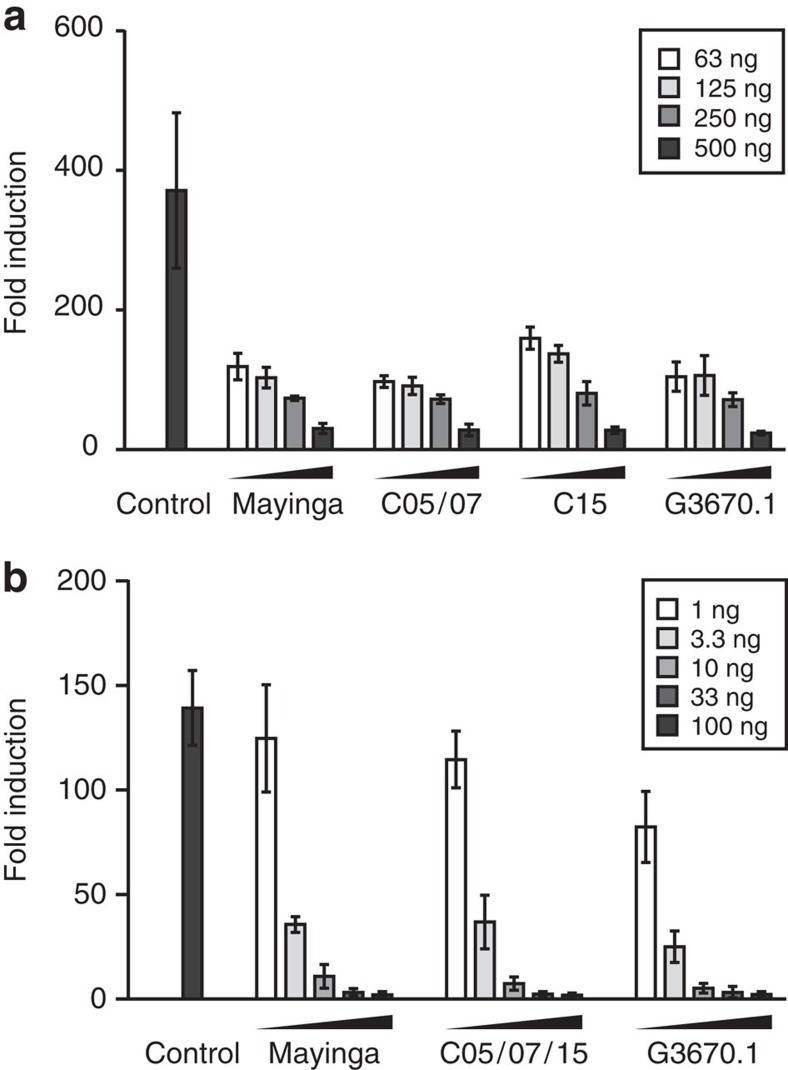
Function of VP35 and VP24 variants in IFN antagonism. (**a**) Relative inhibition of IFN-β production by VP35. 293 cells were transfected with expression plasmids encoding different variants of the EBOV VP35 or empty vector (control), and a luciferase-reporter plasmid under control of an IFN-β promoter. Twenty-four hours later, cells were transfected either with a plasmid encoding the RIG-I CARD domain (stimulated) or with empty vector (unstimulated). After further 24 h, cells were harvested and reporter activity was measured. Fold induction was calculated as the ratio of reporter activity in stimulated to unstimulated cells. The mean and s.d. of three independent experiments are shown. (**b**) Relative inhibition of IFN-β signalling by EBOV protein VP24. 293 cells were transfected with increasing amounts of expression plasmids encoding different variants of the EBOV protein VP24 or empty vector (control), and a luciferase-reporter plasmid under control of an ISRE. Twenty-four hours later, cell medium was replaced with either fresh medium (unstimulated) or medium containing 1,000 I.U. human IFN-β 1a (stimulated). After further 8 h, cells were harvested and reporter activity was measured. Fold induction was calculated as in **a**. The mean and s.d. of three independent experiments are shown.

As a next step, we assessed whether the genetic differences apparent in the Makona variant sequences had any effect on the ability of VP35 to inhibit IFN-β production. To this end, 293 cells were transfected with a reporter plasmid under the control of an IFN-β promoter and with increasing amounts of the different VP35 constructs. At 24 h post transfection, a plasmid expressing the constitutively active CARD domain of RIG-I was transfected into cells to stimulate IFN production^27^. We found that all of the VP35 constructs encoding Makona variant sequences had similar abilities to inhibit IFN-β production (Fig. 3a), as compared with VP35 from the Mayinga isolate, with no statistical differences being detected. This remained true over a range of different expression levels, and thus indicates that these new VP35 Makona variants are as potent as Mayinga VP35 in their ability to inhibit IFN-β production. Further reinforcing this point is the fact that none of the observed genetic differences in Makona isolate VP35 proteins occur in regions known to be involved in antagonism of the IFN response^8^,^28^,^29^,^30^,^31^,^32^,^33^,^34^ (Fig. 1a).

We likewise tested the ability of the VP24 constructs to inhibit the IFN system, this time assaying for the ability of VP24 to suppress IFN-β signalling. To examine this, we transfected 293 cells with a reporter plasmid under the control of an IFN stimulated response element (ISRE) and increasing amounts of the different Makona VP24 variants. After 24 h, cells were stimulated with 1,000 IU human IFN-β to initiate IFN signalling and subsequent induction of the ISRE promoter. The two distinct Makona VP24 sequences examined here were as effective as Mayinga VP24 in inhibiting the IFN-β signalling activity (Fig. 3b) with no statistically significant differences. Also, the mutations observed in the different Makona variant genotypes do not co-localize with regions previously identified as being involved in IFN antagonism^19^,^21^,^34^,^35^,^36^,^37^ (Fig. 1b). Together, this indicates that the sequence variations observed among Makona variants do not affect the ability of these proteins to antagonize IFN signalling.

## Discussion

Our experimental studies have found no evidence for differences in the function of VP35 and VP24 from any of the currently published sequence variants of the Makona variant of EBOV, either among each other or in comparison to the prototype EBOV Mayinga. While EBOV virulence is almost certainly multifactorial in nature^38^ and many of its mechanisms remain incompletely characterized, antagonism of the type I IFN response certainly appears to play a key role^5^. Thus, while we cannot definitively exclude that there might be differences in virulence between Mayinga and Makona EBOVs, our study shows no differences in expression levels, replication and transcription, or IFN antagonism, for Mayinga or Makona VP35 and VP24, and consequently provides no scientific support for claims of heightened virulence of the virus involved in the current outbreak in West Africa. This is in line with epidemiological and case management data, which do not support increased virulence in humans during the current outbreak^39^. The extent of the current outbreak is most likely multifactorial, with the delayed recognition of the outbreak, as well as regional social-cultural aspects, being possible contributing factors.

Also conceptually, hypervirulence, while a common feature of zoonotic pathogens due to a lack of co-evolution with their dead-end hosts, is usually considered maladaptive. Indeed, after switching hosts it is expected that a virus rather attenuates to a lower level of virulence resulting in a longer time window for its transmission, which is limited during debilitating acute infections^40^. A particularly well-characterized example of a hypervirulent virus attenuation following host switching is the classic case of Myxoma virus introduction into Australian rabbits^41^; however, mortality data also suggest that a similar process occurred during the H1N1 Spanish Flu pandemic, with substantial attenuation of the virus occurring within a few years of its introduction into humans^42^.

Finally, it is important to note that despite extensive and on-going human-to-human transmission and the fact that our sequences came from samples spanning 9 months of the outbreak, we observed only three different protein sequences for VP35 and two for VP24. Since submission of this manuscript, additional sequences have continued to be published, including 175 near-complete sequences reported recently by Tong *et al.*^43^. Multiple sequence alignments of these new sequences, in addition to the 108 sequences we initially analysed, revealed only 2 additional non-synonymous changes in VP35 and VP24, which were apparent only in a small subset of samples, and did not coincide with known functional regions. This lack of amino acid sequence diversity speaks against adaptation of these genes in a way that would alter protein function, and is in line with our recent results showing that EBOV in the current outbreak is not subject to accelerated evolution^4^. This is in contrast to speculations, both in the scientific literature as well as the public press, and thus important for the development of rational public health policy during this and future outbreaks. However, given the fact that new sequences continue to be published, it remains important to continue this type of analysis on an on-going basis, to be able to detect putative adaptation events that might occur at later times during the outbreak.

## Methods

### Cloning and mutagenesis

The coding regions of the VP35 genes from EBOV Yambuku isolate Mayinga and EBOV Makona isolates C05 and C15 (accession numbers NC_002549, KP096420 and KP096422, respectively), as well as the coding regions of the VP24 genes from Mayinga and C05, were inserted into pCAGGS (ref. 44) using standard cloning techniques. To generate the VP35 and VP24 genes of the G3670.1 sequence from Sierra Leone (accession number KM034553), point mutations were introduced to the corresponding plasmid-encoded genes of the C05 isolate using site-directed mutagenesis. VP35 proteins encoded by the EBOV Makona isolate C07 and all sequences from Sierra Leone and Mali with the exception of G3680.1 were identical to C05. VP24 proteins encoded by all sequences with the exception of G3680.1 were identical to C05. All plasmids were sequence verified. Primer sequences and detailed cloning strategies are provided in the Supplementary Methods.

### Cell culture

Human embryonic kidney 293 cells (ATCC CRL-1573) were cultured in Dulbecco’s minimal essential medium (Sigma-Aldrich) supplemented with 10% (v/v) foetal bovine serum (heat inactivated at 56 °C for 30 min) (Life technologies), 1% L-glutamine (2 mM) (Life technologies) and 1% penicillin/streptomycin (100 U ml^−1^) (Life technologies) in 5% CO_2_ in a humidified incubator at 37 °C.

### Detection of proteins

VP35 and VP24 were detected by western blot after SDS–PAGE under reducing conditions in a 12% gel. VP35 was detected using a mouse monoclonal anti-EBOV virus Zaire VP35 N terminus (6C5) antibody (1:2,000 dilution)^8^. VP24 was detected with a pooled rabbit anti-peptide antibody (1:2,000 dilution). Donkey anti-mouse and anti-rabbit IgG (Jackson ImmunoResearch) at a 1:50,000 dilution were used as secondary antibodies and detected using SuperSignal West Pico Chemiluminescent Substrate (Thermo Scientific).

### Minigenome analysis

293 cells were seeded in 96-well plates 1 day before transfection for 30–50% confluence at the time of transfection. Initial transfection was performed using TransIT-LT1 (Mirus) according to the manufacturer’s instructions. Each experimental well-received the following plasmids, which have been previously described^17^: pCAGGS-NP (6.9 ng), pCAGGS-VP30 (4.2 ng), pCAGGS-L (55.6 ng) and pCAGGS-T7 polymerase (13.9 ng), as well as pCAGGS-Firefly Luciferase (1.4 ng) for normalization. In addition, each well was transfected with a previously described monocistronic EBOV minigenome plasmid (13.9 ng)^17^ and various amounts of pCAGGS-VP35 (0–55.56 ng) encoding either the VP35 of the Mayinga isolate, the Guinean C05 or C15 isolates of Makona, or the Sierra Leonean Makona sequence G3670.1. Differences in total transfected plasmid mass were equalized by including varying quantities of pCAGGS–green fluorescent protein. Cells were harvested in 100 μl Glo Lysis Buffer (Promega) 48 h after transfection, and 40 μl lysate each were measured for reporter activity using the BrightGlo and RenillaGlo luciferase assay buffer systems (Promega) and a Modulus microplate luminometer (Turner Biosystems) according to the manufacturers’ instructions. Minigenome assays for VP24 function were carried our as described above, but using pCAGGS-VP35-Mayinga (6.9 ng), and varying amounts of a VP24 expression plasmids (0.00–55.56 ng) encoding the VP24 of the Mayinga isolate, the C05 Guinean Makona isolate or the Sierra Leonean Makona sequence G3670.1.

### IFN-β production inhibition assay

IFN production assays were performed as previously described^45^. 293 cells were seeded in 12-well plates 1 day before transfection for 30–50% confluence at the time of transfection. Initial transfection was performed using TransIT-LT1 according to the manufacturer’s instructions. Plasmids transfected were a construct encoding firefly luciferase under the control of the IFN-β promoter (pI125luc, 250 ng), a construct encoding *Renilla* luciferase (pCAGGS-hrluc, 50 ng) for normalization and pCAGGS-VP35 (62.5–500 ng, in twofold dilutions). Wells receiving <500 ng of the VP35 construct received empty pCAGGS to compensate for differences in total plasmid mass. Twenty-four hours later, a second transfection was performed, again using TransIT-LT1, with 200 ng of a pEF construct encoding amino acids 1–200 of the human RIG-I CARD domain, which had been constructed from pEF-BOS RIG-I kindly provided by Kate Fitzgerald (Addgene plasmid # 27236)^46^, using the Quick Change Lightning site-directed mutagenesis kit (Agilent). Cells were harvested in 100 μl Glo Lysis buffer, and reporter activity in 40 μl lysate each was measured 24 h after the second transfection using the BrightGlo and RenillaGlo reagents and a Modulus microplate luminometer.

### IFN-β signalling inhibition assay

293 cells were seeded in 12-well plates 1 day before transfection for 30–50% confluency at the time of transfection. Transfection was performed using TransIT-LT1 according to the manufacturer’s instructions. Plasmids transfected included 250 ng firefly luciferase in a pISRE reporter plasmid (Agilent)^47^, 50 ng pCAGGS-hrluc for normalization and 1–100 ng (in threefold dilutions) pCAGGS-VP24, with wells receiving <100 ng VP24 balanced by empty vector. Twenty-four hours later, 1,000 IU human IFN-β 1a (PBL Assay Science) were added. Cells were harvested and reporter activity was measured 8 h after IFN application as described for the IFN production assay.

### Statistical analysis

Differences in reporter activity in the presence of Mayinga or the various Makona variant proteins at each transfection amount were calculated and analysed by one-way analysis of variance followed by Tukey’s multiple comparison test using Prism 6.04.

## Additional information

**How to cite this article:** Dunham, E. C. *et al.* Assessing the contribution of interferon antagonism to the virulence of West African Ebola viruses. *Nat. Commun.* 6:8000 doi: 10.1,038/ncomms9000 (2015).

## Supplementary Material

Supplementary Information Supplementary Figure 1 and Supplementary Methods
