# Supplementary material for: Assessing the contribution of interferon antagonism to the virulence of West African Ebola viruses
Source: Nat Commun. 2015 Aug 5;6:8000. doi: 10.1038/ncomms9000 (PMC4527089; doi:10.1038/ncomms9000)
Supplement: Supplementary Information — Supplementary Figure 1 and Supplementary Methods [file ncomms9000-s1.pdf]

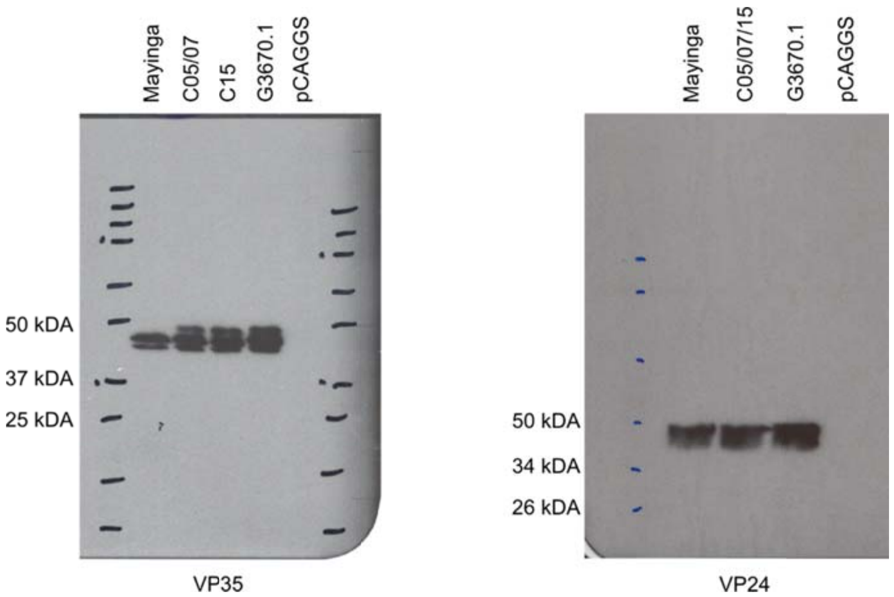

Supplementary Figure 1: Full Western blots of VP35 and VP24 expression. Full blots, portions of which are presented in Figure 1C, are shown.

Supplementary Methods

Cloning of VP35 and VP24 variants for Mayinga, C05, and C15 isolates: All VP35 variants were inserted into the pCAGGS vector using the restriction enzymes NotI and SacI. All VP24 variants were inserted with the enzymes XhoI and BglII. Inserts were cloned either from plasmids (Mayinga sequences) or cDNA (C05 and C15 sequences). PCR was carried out using iProof Polymerase (BioRad) according to manufacturer's instructions. Primers used were as follows:

VP35_C05_C15_May_NotI_fwd: GACTCAGCGGCCGCATGACAACTAGAACAAAGGGCAGG

VP35_C05_C15_May_SacI_rev: GTCCAGGTCGAGCTCGTCAAATTTTGAGTCCAAGTGTTTTACCATC

VP24_C05_XhoI_fwd: CATGATCTCGAGATGGCCAAAGCTACGGGACG

VP24_May_XhoI_fwd: CATGATCTCGAGATGGCTAAAGCTACGGGACG

VP24_C05_BglII_rev: AGCACGAGATCTGTTAGATAGCAAGAGAGCTATTGAATTC

VP24_May_BglII_rev: AGCACGAGATCTGTTAGATAGCAAGAGAGCTATTAAATTC

Cloning of G3670.1 sequences for VP35, VP24: To create plasmids encoding for VP35 and VP24 from the Sierra Leone G3670.1 sequence, the plasmids encoding the C05 sequences for each of these proteins were used for site-directed mutagenesis using the following primers:

VP35-T3388G-fwd: CCAATGAAGCCAATGTTTGTCCTACCTCCTCAAAACTATGA

VP35-T3388G-rev: TCATAGTTTTGAGGAGGTAGGACAAACATTGGCTTCATTGG

To achieve the single amino acid change differentiating the G3670.1 sequence from that of C05, the gene was amplified in two parts using VP24_C05_XhoI_fwd and VP24-A10801G-rev (CCGTGCATGCGTCTCTAACCAACGACAGCATTTTTAGGCTC), and VP24_C05_BglII_rev and ED16_VP24-A10801G-fwd (CATGGCATGCGTCTCAGGTTCGATCCAATATTCTCAAGTTTATTAAC), which were joined after BsmBI digest, and cloned into pCAGGS via XhoI and BglII.

Primers used for creation of RIG-I CARD domain plasmid: The coding region between amino acids 200 and 925 was deleted from a RIG-I plasmid using the Quick Change Lightning site-directed mutagenesis kit (Agilent) according to manufacturer’s instructions. The following primers were used: CTTCCTTCAATGGATCCTAAGATCTTCTGTTTCAACATCTTTTATACCTT, AAGGTATAAAAGATGTTGAAACAGAAGATCTTAGGATCCATTGAAGGAAG.
